# Increased Degenerative Biomarkers in Females with Patellofemoral Pain: A Cross-Sectional Analysis with 6-Month Progression

**DOI:** 10.3390/diseases13050155

**Published:** 2025-05-17

**Authors:** Lori A. Bolgla, Tiana V. Curry-McCoy, Maya Giddens, Madelyn Overton, Bryaunna Barrera, Jasmine Crockett, Monte Hunter

**Affiliations:** 1 Department of Physical Therapy, College of Allied Health Sciences, Augusta University, Augusta, GA 30912, USA; 2 Department of Undergradaute Health Professions-Clinical Laboratory Science Program, College of Allied Health Sciences, Augusta University, Augusta, GA 30912, USA; tcurrymccoy@augusta.edu (T.V.C.-M.); maya.giddens@wellstar.org (M.G.); madelyn.overton@wellstar.org (M.O.); bryaunna.barrera@wellstar.org (B.B.); jasmine.crockett@wellstar.org (J.C.); 3 Department of Orthopaedic Surgery, Medical College of Georgia, Augusta University, Augusta, GA 30912, USA; mohunter@augusta.edu

**Keywords:** anterior knee pain, cartilage, prognosis, collagen, degeneration, prospective

## Abstract

Background/Objectives: Patellofemoral pain (PFP) is considered a risk factor for knee osteoarthritis (OA) onset. The purpose of this study was to compare degenerative biomarkers in females with and without PFP and to determine changes in these levels, along with pain and function, over 6 months. Methods: All subjects received a knee x-ray to ensure that none had degenerative changes. Urine and serum were collected and analyzed for C-telopeptide fragments of type II collagen (CTX-II) and C-propeptide II (CP-II); these were then expressed as a cartilage degradation: cartilage synthesis ratio (CTX-II:CP-II). Subjects with PFP rated pain using a 10 cm visual analog scale, and function using the Knee injury and Osteoarthritis Outcome Scores-Patellofemoral (KOOS-PF) questionnaire. Subjects with PFP were tested at baseline and at 6 months. Results: Females with PFP had higher levels of CTX-II:CP-II than controls (*p* < 0.001) and these remained elevated at 6 months (*p* = 0.82). Females with PFP reported similar levels of pain (*p* = 0.30) but higher function at 6 months (*p* = 0.002). However, the 9.0-point increase in KOOS-PF values did not exceed the minimum important change. Conclusions: Females with PFP but no evident structural changes had more elevated biomarkers than controls. This finding suggests that this cohort may have excessive cartilage turnover which may contribute to knee OA.

## 1. Introduction

Patellofemoral pain (PFP) is one of the most common, yet challenging, knee problems to manage. Estimates for the prevalence of PFP among those with knee pain complaints have ranged from 25% to as high as 45% [1,2]. Researchers believe that PFP may be a precursor to knee osteoarthritis (OA) [3,4,5]. PFP and knee OA have similar features, and females are more likely than males to develop each pathology [2,6]. More concerning are the negative effects that PFP and knee OA can have on function, quality-of-life, physical activity, and mental health [7,8,9,10,11].

PFP is a diagnosis made primarily from self-reported pain either around or behind the patella during activities that require loading on a flexed knee (e.g., running, jumping, climbing stairs, and squatting). However, radiographs and magnetic resonance imaging (MRI) provide little, if any, information regarding degenerative cartilage changes [12,13], presenting a challenge to PFP diagnosis. Poole et al. [14] have stated that degenerative changes may not become evident for over 20 years.

Detection of knee degenerative changes typically has focused on anatomic derangements and not the underlying molecular pathogenesis [15]. Furthermore, pain and anatomic changes are not necessarily associated, especially early in the disease process [16,17]. An understanding of molecular causes may help identify early patellofemoral joint degeneration and support early intervention. Lotz et al. [18] have emphasized the importance of biomarkers in preventing and managing knee OA. We believe that biomarkers may provide important information regarding PFP as well. While PFP is often attributed to biomechanical factors, underlying inflammatory processes may contribute to early joint degeneration. Given the proven utility of biomarkers in knee OA, identifying similar biomarkers in individuals with PFP may enable early detection of joint changes and identification of patients at risk of progression. Early identification supports the importance of early rehabilitation (e.g., strengthening, stretching, movement retraining, and activity modification) to prevent PFP from transitioning from an acute, localized problem to a chronic one [19]. Moreover, healthcare use, costs, and odds of recurrence are lower among those who seek physical therapy services close to the initial time of diagnosis [19].

To date, only two studies have examined cartilage degradation biomarkers in individuals with PFP. Murphy et al. [20] reported increased levels of serum cartilage oligomeric matrix protein (COMP), a biomarker indicative of cartilage degradation, in subjects with (*n* = 18) and without (*n* = 14) chondromalacia patella (another term used to describe PFP). However, Bolgla et al. [21] found no differences in C-telopeptide fragments of type II collagen (CTX-II) between females with (*n* = 18) and without (*n* = 12) PFP. Limitations existed in both studies. First, they both used a single biomarker that may not be robust enough to characterize PFP pathophysiology [22,23]. Second, these works were cross-sectional and did not examine changes in biomarker levels over time in those with PFP. Finally, while these works examined pain levels, they did not include any reliable, validated patient-reported outcome measures related to function and quality-of-life.

The purpose of this study was two-fold. The first purpose was to compare a cluster of biomarkers in females with and without PFP. The second purpose was to determine changes in biomarker levels, pain, and function/quality-of-life in females with PFP over a 6-month period. We hypothesized the following: (1) females with PFP would have significantly higher biomarker levels than controls; and (2) females with PFP would continue to have elevated biomarker levels, pain levels, and lower function/quality-of-life at 6 months.

## 2. Materials and Methods

### 2.1. Subjects

**Figure 1:**
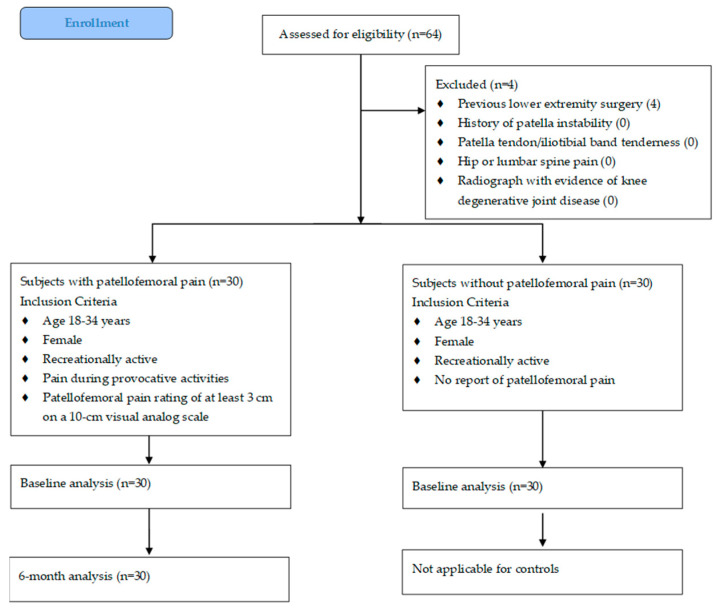
CONSORT diagram for subject enrollment.

An a priori power analysis was conducted using G*Power (v3.1.9.7) [24]. Based on a large effect size (d = 0.80), α = 0.05 and β = 0.20, the analysis suggested a minimum of 26 subjects were needed to obtain statistical power. Subjects were recruited from the greater Augusta, GA area via word-of mouth and recruitment flyers. Only females were recruited due to naturally occurring sex differences (e.g., hormonal influences and known sex differences in extracellular vesicle protein cargo in synovial fluid in knee OA) in the biomarkers [25,26]. Thirty females with PFP and thirty controls participated (Figure 1). Subject age ranged from 18 to 33 years because the incidence of knee OA is most likely to occur after 40 years [27]. Inclusion and exclusion criteria were as previously reported [28]. Briefly, participants were classified as recreationally active if they had engaged in physical activity for at least 30 min on a minimum of three days per week over the previous six months. Individuals with PFP were required to meet additional inclusion criteria specific to anterior knee symptoms: (1) a minimum pain rating of 3 out of 10 on a visual analog scale (VAS) during daily or recreational activities (e.g., walking, running, climbing stairs, or squatting) within the past week; (2) symptom duration of at least four weeks with insidious onset; and (3) pain reproduced by at least three of the following: physical activity, prolonged sitting, climbing stairs, or squatting. No participants with PFP had received rehabilitation prior to or during study participation. Exclusion criteria included the following: a history of lower-extremity surgery or major injury; recurrent patellar dislocation or subluxation; tenderness over the patellar tendon or iliotibial band; and referred pain from the hip or lumbar spine. Five individuals with PFP reported bilateral symptoms. Participants were enrolled consecutively as they met eligibility criteria, and all signed an informed consent document approved by the Augusta University Institutional Review Board (1480126). All aspects of the study were conducted in accordance with the Declaration of Helsinki.

To ensure that subjects did not have any evident joint degenerative changes to the patellofemoral joint, they received an x-ray (sagittal plane and sunrise views) prior to data collection. All images were interpreted by an experienced orthopedic surgeon (D.M.H.) who was blinded to each subject’s group assignment. No subject showed signs of knee (tibiofemoral) or patellofemoral joint degradation.

### 2.2. Cartilage Biomarker Sample Collection

Based on prior works, we assessed a cluster of cartilage biomarkers [22]. CTX-II, a cartilage degradation biomarker, has been known to be elevated in individuals with radiographically defined knee OA [22,29]. More importantly, CTX-II levels can decrease with rehabilitation [30,31]. The other biomarker, C-propeptide II (CP-II), is a type II synthesis cartilage biomarker whose levels have been shown to be higher in those without knee OA [22]. These biomarkers were selected based on evidence showing that they could be used to differentiate between individuals with knee pain who either have or do not have radiographic signs of knee OA [22]. Although a prior study examined COMP [20], we did not include because COMP cannot be used to discriminate between painful knees with or without radiographic knee OA [22].

#### 2.2.1. Urine Analysis

Subjects with and without PFP provided an early-morning, second-void urine sample, which was processed and stored at −80 °C until the time for analysis [30]. All data were deidentified and analyzed in duplicate using a commercially available enzyme-linked immunosorbent assay (ELISA) based on a mouse monoclonal antibody raised against the EKGPDP sequence of human CTX-II (Urine CartiLaps^®^ ELISA (CTX-II); BioVendor, LLC; Asheville, NC, USA). CTX-II was corrected for urinary creatinine concentration using the following formula per manufacturer’s instruction: corrected CTX-II (ng/mmol) = [1000 × Urine CartiLaps (ng/mL)]/creatinine (mmol/L). All procedures were conducted in accordance with the manufacturer’s instructions. Subjects with PFP returned to the laboratory for repeat measures 6 months later. Baseline and 6-month samples were analyzed at the same time.

#### 2.2.2. Serum Analysis

Blood samples were collected in serum blood collection tubes and placed in a vertical position for 30 min. Afterward, samples were centrifuged in a swinging-bucket rotor at room temperature for 20 min at 1200× *g*, in accordance with manufacturer’s instructions. Samples were stored at −80 °C until the time for analysis. All data were deidentified and analyzed in triplicate using a commercially available ELISA based on a primary rabbit polyclonal antiserum specific for CP-II (CP-II ELISA; IBEX Pharmaceuticals; Montreal, QC, Canada). CP-II levels were expressed in ng/L. All procedures were conducted in accordance with the manufacturer’s instructions. Subjects with PFP returned to the laboratory for repeat measures 6 months later. Baseline and 6-month samples were analyzed at the same time.

#### 2.2.3. Data Reduction

CTX-II and CP-II data were log-transformed to minimize the influence of outliers [30]. The log transformation process converted CTX-II and CP-II to unitless measures. The biomarkers were expressed as a ratio by dividing CTX-II by CP-II (CTX-II:CP-II) [22]. A higher value suggested greater cartilage degradation than cartilage synthesis.

### 2.3. Pain and Function/Quality-of-Life Measures

#### 2.3.1. Pain Measures

Subjects with PFP used a 10 cm VAS to rate their pain during activity over the previous week. Pain during activity over the previous week was used due to its established reliability, responsiveness, and validity for those with PFP [32]. The extreme left side of the VAS represented “no pain” and the extreme right side represented “worst pain imaginable”. Subjects drew a perpendicular line on the VAS to show the position that best described pain during activity over the previous week. The distance from the extreme left side of the VAS to the perpendicular line drawn by the subject was measured to the nearest 1/10th of a cm and used for statistical analysis. Subjects with PFP returned to the laboratory for repeat measures 6 months later.

#### 2.3.2. Function/Quality-of-Life Measures

Subjects with PFP completed the Knee injury and Osteoarthritis Outcome Scores-Patellofemoral (KOOS-PF) subscale at baseline and 6 months. The KOOS-PF is an 11-item patient-reported outcome measure designed to assess function and quality-of-life in individuals with PFP [33]. Hoglund et al. [34,35] recommend using the KOOS-PF for clinical and research purposes based on its established content validity, reliability, and responsiveness. The KOOS-PF is scored on a 0–100 scale. A higher value suggests greater function and quality-of-life. The minimal important change (MIC) for the KOOS-PF is 14.2 points [33]. Subjects with PFP returned to the laboratory for repeat measures 6 months later.

### 2.4. Statistical Analysis

Parametric tests were used because CTX-II:CP-II values were log-transformed to reduce skewness. In addition, each group included 30 subjects, meeting the assumption of the central limit theorem. Means and standard deviations were calculated for all dependent measures. An independent *t*-test was used to compare CTX-II, CP-II, and CTX-II:CP-II levels in females with and without PFP at baseline. A separate independent *t*-test was used to compare the 6-month CTX-II:CP-II levels in females with PFP to control levels collected at baseline. Paired *t*-tests were used to compare baseline and 6-month CTX-II:CP-II, VAS, and KOOS-PF values for females with PFP. Effect sizes (e.g., Cohen’s d) were also calculated. The level of significance was set to 0.05. All analyses were conducted using IBM SPSS Statistics for Windows, Version 28 (IBM Corp., Armonk, NY, USA).

## 3. Results

### 3.1. Comparison of CTX-II, CP-II, and CTX-II:CP-II Levels in Females with PFP and Controls

**Figure 2:**
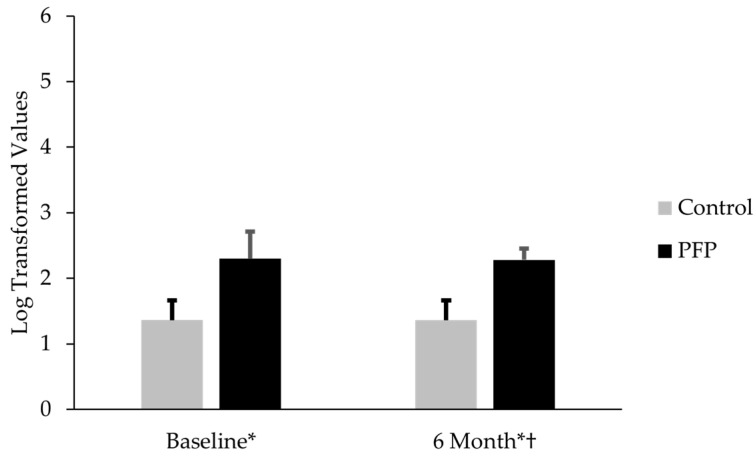
Comparison of ratio of C-telopeptide fragments of type II collagen to C-propeptide II (CTX-II:CP-II) between controls and subjects with patellofemoral pain (PFP) at baseline and 6-month follow-up. Baseline levels for controls were compared to 6-month levels for subjects with PFP. * CTX-II:CP-II was significantly lower in controls than in subjects with PFP at baseline and 6-month follow-up (*p* < 0.001). † CTX-II:CP-II was similar in subjects with PFP at baseline and 6-month follow-up (*p* = 0.82).

Thirty females with PFP participated along with thirty controls. Subjects were similar with respect to age, mass, and height (Table 1). When analyzed separately, females with PFP had similar CTX-II levels (mean difference = 0.02; t(58) = 0.20; *p* = 0.84; Cohen’s d = 0.05) to controls. CP-II levels in females with PFP were 150% lower than controls (mean difference = 0.92; t(58) = 29.8; *p* < 0.001; Cohen’s d = 7.7) (Table 2). Females with PFP had CTX-II:CP-II levels that were 69.1% higher than controls (mean difference = 0.94; t(58) = 10.1; *p* < 0.001; Cohen’s d = 2.6) (Figure 1). The CTX-II:CP-II values for subjects with PFP at 6 months were 67.6% higher than the controls’ baseline levels (mean difference 0.92; t(58) = 14.6; *p* < 0.001; Cohen’s d = 3.8) (Figure 2).

### 3.2. Comparison of CTX-II:CP-II, VAS, and KOOS-PF Values for Females with PFP at Baseline and 6-Month Follow-Up

**Figure 3:**
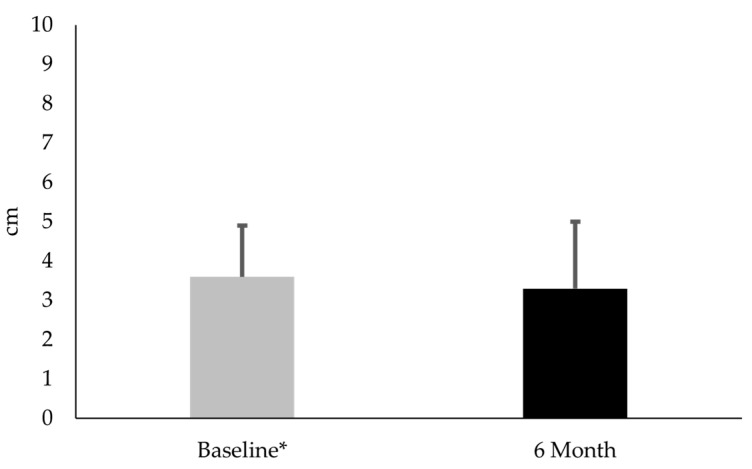
Comparison of 10 cm visual analog scale (VAS) measures for average pain over the prior week during activity for subjects with patellofemoral pain at baseline and 6-month follow-up. * VAS measures were similar at baseline and 6-month follow-up (*p* = 0.30).

**Figure 4:**
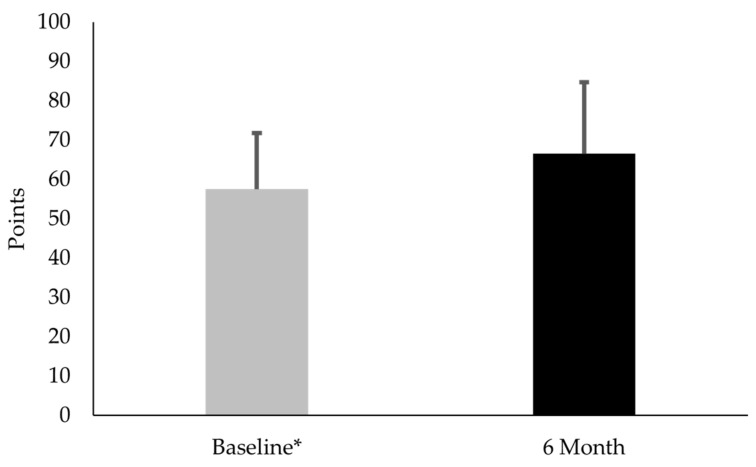
Comparison of Knee injury and Osteoarthritis Outcome Scores-Patellofemoral (KOOS-PF) subscale points for subjects with patellofemoral pain at baseline and 6-month follow-up. * KOOS-PF scores were lower at baseline, compared with 6-month follow-up (*p* = 0.002).

Thirty females with PFP participated in this part of the study. No difference existed between CTX-II:CP-II levels at baseline and 6-month follow-up (mean difference −0.02; t(29) = 0.23; *p* = 0.82; Cohen’s d = 0.1) (Figure 2). No difference existed in VAS between baseline and 6-month follow-up (mean difference −0.38; t(29) = 1.1; *p* = 0.30; Cohen’s d = 0.2) (Figure 3). KOOS-PF scores were 15.7% higher at 6 months than at baseline (mean difference 9.0; t(29) = 3.36; *p* = 0.002; Cohen’s d = 0.6) (Figure 4).

## 4. Discussion

PFP is a common knee problem which is believed to be a risk factor for the development of knee OA. A challenge posed by PFP is that it is diagnosed based on subjective signs and symptoms, and imaging provides limited information. Cartilage biomarkers may provide important insight because they may detect biological changes prior to structural changes being evident with imaging [15]. The current study is the first to examine a cluster of degenerative cartilage biomarkers in females with and without PFP.

### 4.1. Comparison of CTX-II, CP-II, and CTX-II:CP-II Values for Females with and Without PFP at Baseline

Our results supported the first hypothesis because females with PFP had significantly higher CTX-II:CP-II levels than controls. This finding is clinically important because it contributes to the current body of knowledge that PFP may contribute to knee OA onset [4]. Understanding possible degenerative changes has been challenging, given the limited usefulness of imaging [12,13]. Moreover, degenerative changes can have a lengthy latency period and may not be evident for over 20 years following PFP onset [14].

Cibere et al. [22] examined 10 biomarkers in 201 individuals (ages 40–79 years; 105 (52.2%) females) with knee pain. They found that individuals with radiographically defined knee OA were 3.4 times more likely to have elevated CTX-II:CP-II levels than those with pain but no degenerative changes. Notably, all subjects in the current study had radiographs that showed no signs of degenerative changes; however, those with PFP had elevated CTX-II:CP-II levels. This finding suggests that CTX-II:CP-II may detect excessive cartilage turnover prior to visible structural changes.

Distinct findings were obtained when analyzing CTX-II and CP-II separately. Our preliminary study, currently the only published work to compare CTX-II levels between females with and without PFP, found no significant between-group differences [21]. Similarly, the present study detected no differences in CTX-II, the cartilage degradation biomarker. This consistent finding highlights a potential limitation in using a single biomarker to capture the complexity of PFP pathophysiology. No previous study has investigated CP-II, a cartilage synthesis biomarker, in individuals with PFP. Our data revealed significantly lower CP-II levels in females with PFP, suggesting reduced cartilage regeneration. When considered together, the elevated CTX-II:CP-II levels in the PFP group suggested an imbalance in cartilage turnover, favoring degradation over synthesis. This ratio may provide a more comprehensive assessment of cartilage metabolic activity than either biomarker alone. These findings highlight the importance of utilizing a biomarker cluster to better characterize the molecular changes underlying PFP and thus identify individuals at greater risk of cartilage degeneration [22].

In summary, CTX-II:CP-II may serve as a biomarker that can identify those with PFP who may be at higher risk for developing knee OA. However, additional studies are needed to establish normative values and determine clinically meaningful elevated values across age and sex. Long-term longitudinal studies also are needed to determine changes in CTX-II:CP-II over time in combination with MRI changes.

### 4.2. Comparison of CTX-II:CP-II, VAS, and Koos-PF Values for Females with PFP at Baseline and 6 Months

A strength of this study was its prospective nature, which allowed natural changes over time to be assessed without intervention. Most importantly, the females with PFP were able to continue with their usual activities. At the end of this period, their CTX-II:CP-II levels remained significantly higher than those for controls at baseline. This pattern of persistent biomarker elevation may indicate ongoing abnormal cartilage metabolism that could lead to joint deterioration. Additional longitudinal studies are needed to make this determination.

Regarding the clinical measures, females with PFP had similar VAS scores at 6 months. This finding suggested that symptoms associated with PFP remained relatively stable. Interestingly, these subjects had an average 9.0-point increase in KOOS-PF values. While this change was statistically significant, it did not exceed the MIC, the minimal amount of change deemed meaningful to a patient, and thus highlighted an important distinction between statistical significance and clinical meaningfulness [36]. In brief, an increase from a statistical standpoint did not necessarily translate into a meaningful difference in function and quality-of-life from the subjects’ perspective. The increased KOOS-PF scores suggested that females with PFP experienced subtle increases in function without any changes in cartilage degradation biomarker levels or pain.

### 4.3. Clinical Implications

Our findings are clinically important because they provide additional evidence that PFP is not a benign condition but one of ongoing pathology. They suggest that females with PFP and elevated biomarkers may have experienced early biological changes that could lead to patellofemoral joint degradation. This pattern highlights the importance of early detection and additional information regarding prognosis. For example, examining CTX-II:CP-II levels with other clinical measures like lower-extremity kinematics, strength, and flexibility may help identify females with PFP who may be at a higher risk for a poor outcome. Identifying this cohort in a timely manner could lead to early rehabilitation that reduces CTX-II levels [30,31].

### 4.4. Limitations

This study has limitations that deserve consideration. While one strength of this study was its use of a “wait-and-see” period, the duration of this period was likely not sufficient to detect subtle changes. This study did not include a treatment group, which limits the generalizability of findings to individuals receiving treatment. Future studies should examine changes in CTX-II:CP-II levels following a course of rehabilitation. We purposefully only included females due to naturally occurring differences in biomarker levels, precluding the ability to generalize findings to males with PFP. Moreover, we did not collect data regarding menstrual cycle, diet, or activity. These factors, especially hormonal fluctuations, could have affected biomarker levels. Future studies should consider these influences. Although cartilage biomarkers are useful for monitoring disease progression, they are only indirect measures. Longitudinal studies are needed to simultaneously examine biomarker and structural (via MRI) changes over time. Finally, we limited our age range to young adults; it remains unknown whether other age ranges, especially adolescents, have elevated CTX-II:CP-II levels.

## 5. Conclusions

This study was unique because it was the first to examine a cluster of cartilage degradation biomarkers in females with PFP. We found that females with PFP had elevated CTX-II:CP-II levels, compared with controls, and remained elevated at 6 months. Our results add to the current body of evidence suggesting a link between PFP and knee OA onset. This information also highlights the possible use of this biomarker for prognostic purposes. Future works are needed to determine the long-term effects of elevated CTX-II:CP-II levels on joint health and whether these levels decline with rehabilitation.

## Figures and Tables

**Table 1:** Means ± standard deviations for subject demographics for females with patellofemoral pain (PFP) and controls.

	PFP	Controls	*p*-Value
Age, y	22.9 ± 2.9	22.6 ± 3.3	0.75
Mass, kg	71.9 ± 17.3	65.9 ± 16.9	0.19
Height, cm	168.3 ± 7.1	165.9 ± 7.2	0.20

**Table 2:** Means ± standard deviations for CTX-II and CP-II for females with patellofemoral pain (PFP) and controls at baseline. All values are unitless log-transformed measures.

	PFP	Controls	*p*-Value
CTX-II	2.9 ± 0.4	2.9 ± 0.3	0.91
CP-II	0.6 ± 0.1	1.5 ± 0.1	<0.001

## Data Availability

Data from this study are available on request to the corresponding author.
